# EFFECTIVENESS OF BEHAVIOURAL MEDICAL REHABILITATION UNDER REALLIFE CONDITIONS IN GERMANY: A PROPENSITY-SCORE MATCHED ANALYSIS

**DOI:** 10.2340/16501977-jrm.v53.469

**Published:** 2021-10-21

**Authors:** Miriam MARKUS, Annemarie EUHUS, Matthias BETHGE

**Affiliations:** Institute for Social Medicine and Epidemiology, University of Lübeck, Lübeck, Germany

**Keywords:** rehabilitation, programme evaluation, mental health comorbidity, work ability, health service research

## Abstract

**Objectives:**

In Germany, behavioural medical rehabilitation programmes have been implemented for patients with musculoskeletal disorders and additional mental health comorbidity. The aim of this cohort study is to assess the relative effectiveness of behavioural medical rehabilitation under real-life conditions.

**Design:**

Participants received either a common or behavioural medical rehabilitation programme. Propensity score matching was used to provide balanced samples of both groups (German Clinical Trials Register: DRKS00016404).

**Participants:**

A total of 360 patients treated in behavioural medical rehabilitation were compared with 360 matched controls. The mean age of study participants was approximately 53.5 years (standard deviation (SD)=7.0 years) and 74.0% were women.

**Results:**

No significant and clinical meaningful differences were found in return to work, applications for disability pension, and the number of patients receiving social security benefits in the year after rehabilitation. However, participants in behavioural medical rehabilitation reported better self-rated work ability, physical functioning and self-management skills, and decreased pain disability and fear-avoidance beliefs 10 months after rehabilitation. Standardized effect sizes were between 0.13 and 0.22.

**Conclusion:**

Behavioural medical rehabilitation had no clinical meaningful effect on maintaining and restoring work ability. However, behavioural medical rehabilitation affected pain and disease management skills 10 months after completing the rehabilitation programme.

LAY ABSTRACT

Mental health comorbidity increases the risk for patients with musculoskeletal disorders to receive a disability pension despite participating in a multimodal rehabilitation programme. In Germany, behavioural medical rehabilitation programmes were therefore developed for people who are affected from both musculoskeletal and mental health disorders. These programmes integrate additional psychological treatment into the standard multimodal rehabilitation approach. To test whether these programmes are more effective in enabling a stable return to work and improving self-rated health, this study compared 360 patients treated in behavioural medical rehabilitation with 360 comparable controls treated in conventional medical rehabilitation. The results indicate that participants could benefit more from a behavioural medical rehabilitation programme. Patients did not return to work significantly more often. However, they reported better self-rated work ability, better pain and health-related outcomes, and fewer days with unemployment benefits. In conclusion, more consistent implementation of the programme offers the opportunity to further improve these outcomes.

Worldwide, hundreds of millions of people are affected by diseases of the musculoskeletal system, such as arthrosis or rheumatic diseases, or injuries following accidents (1). The most common, however, is back pain. Its lifetime prevalence is approximately 85% and, within any 12-month period, 3 out of 4 people experience back pain (2). In 2018, musculoskeletal disorders were the third most frequent reason for receiving disability pension and made up 12.9% of all approved disability pensions in Germany (3).

The aims of medical rehabilitation programmes in Germany are to enable stable participation in working life and to prevent the transition of sickness absence due to illness into permanent work disability. However, it was found that mental health and back pain are closely linked, and patients with musculoskeletal disorders in medical rehabilitation have a 1.6-times higher risk of receiving a disability pension if they also have a mental health disorder (4, 5). In order to achieve the aims of medical rehabilitation, and also in cases of mental health comorbidity, the early identification and treatment of mental health impairments are necessary (6).

With behavioural medical rehabilitation (BMR), a programme has been developed that offers a multidisciplinary, behavioural medical treatment approach for people with both physical and mental health complaints. This rehabilitation programme integrates psychological treatment in individual and group interventions into the standard treatment concept. Unlike common medical rehabilitation (MR), which is usually granted for 3 weeks, BMR is initially granted for 4 weeks by the Federal German Pension Insurance. BMR includes the following additional behavioural medical therapy modules: psychological therapy group, individual psychological consultations, relaxation training, exercise group, and initial and final consultation in exercise therapy. BMR programmes are conducted as a group intervention (7).

Controlled and randomized controlled trials compared behavioural medical rehabilitation programmes with common medical rehabilitation programmes and showed more favourable outcomes in depression, pain-related coping strategies, pain and self-reported health in participants in behavioural medical rehabilitation programmes (8–10). A multicentre study with 4 rehabilitation centres with a treatment focus on behavioural medical rehabilitation compared psychological pain competence training as part of the behavioural medical rehabilitation for patients with chronic back pain with combined pain competence and depression prevention training. This study showed long-term effects in favour of rehabilitation with combined pain and depression management training. After 1 year, patients had a significantly stronger improvement in general and psychological work ability and a significant reduction in pain-related days of sick leave (11).

Evidence of efficacy under controlled conditions is a necessary, but not sufficient condition for ensuring that the intervention also leads to more favourable and comparable results in real-world settings. Effectiveness can be reduced due to shortcomings in the selection of patients and treatment fidelity (12). To date, there is no clear evidence of the effectiveness of BMR implemented in real-life care. Therefore, the aim of this study is to evaluate whether BMR improves outcomes compared with common medical rehabilitation under real-life conditions after 10 months, as well as in the year after rehabilitation. This paper was written according to the Transparent Reporting of Evaluations with Nonrandomized Designs (TREND) statement (13).

## METHODS

### Study design

This was a cohort study under real-life conditions. Data were initially collected during the evaluation of work-related medical rehabilitation (14, 15). In 2 parallel groups, participants received either a BMR or a common medical rehabilitation programme that was performed between March and September 2016. Although the need for BMR is far greater, most people are still treated in common medical rehabilitation. Therefore, comparable controls were available in the much larger pool of patients who received a common medical rehabilitation programme. Propensity score matching was used to identify a similar patient from the pool of patients treated in common medical rehabilitation for every individual treated in BMR. Baseline data were assessed after approval of the rehabilitation by the Federal German Pension Insurance, but before the programme started. Follow-up data were assessed by questionnaire 10 months after completing the rehabilitation programme. Furthermore, data on the treatments provided from the rehabilitation discharge letters were used. Details of the participant’s employment status after finishing the rehabilitation programme were provided by the Federal German Pension Insurance. These data refer to the entire year 2017. Since rehabilitation was performed between March and September 2016, the start of the follow-up period for employment data ranged from 3 months after completion of the rehabilitation programme for patients with rehabilitation in September 2016 to 9 months for patients with rehabilitation in March 2016 (median 7 months). Due to the observational design, no one was blinded before, during or after the trial.

### Treatment

Participants in the intervention group received a BMR programme according to the guideline for BMR (7). In comparison with common medical rehabilitation, BMR includes additional behavioural medical therapy modules that are primarily focused on pain management, dealing with pain-related behaviour and mental health comorbidity. The behavioural medical core therapy modules are: psychological therapy group (at least 8 h per rehabilitation programme), individual psychological consultations (at least 100 min), relaxation training (at least 4 h), exercise group (20 h), and initial and final consultation in exercise therapy (50 min). This results in an additional behavioural medical therapy dose of at least 34.5 h per rehabilitation programme.

In the psychological therapy group, disease management skills and the basics of the relationship between the disease, body, mind and behaviour are taught. Individual psychological consultations usually take place at the beginning and end of rehabilitation in order to define therapy goals and measure the change of symptoms. The relaxation training serves to teach a relaxation method (progressive muscle relaxation or autogenic training) within the therapy group. The exercise group is intended to give the rehabilitants a positive approach to exercise in everyday life again despite their functional limitations and to support their self-management skills. Through experienceoriented body work and everyday forms of movement, coordination and physical skills should be improved. The initial and final consultation in exercise therapy serves to agree on individual goals with concrete exercise goals and to evaluate the individual rehabilitation outcome.

### Controls

Participants in the control group received a common medical rehabilitation without an explicit behavioural medical focus. Common medical rehabilitation was conducted according to current treatment standards and guidelines for the rehabilitation of patients with musculoskeletal disorders. The daily amount of therapy was usually 3–4 h and included sports and exercise therapy, physiotherapy, massages and other physical therapies, as well as social and psychological counselling, patient education, pain management and relaxation training.

### Setting and participants

The study was conducted in 92 rehabilitation centres. Of these, 10 centres provided BMR. Inclusion criteria were: rehabilitation patients with chronic musculoskeletal disorders, mostly chronic back pain (90%), aged 18–65 years. The rehabilitation programme was granted by the Federal German Pension Insurance. The allocation to a behavioural or common medical rehabilitation programme was determined by the socio-medical service of the Federal German Pension Insurance, based on the available findings and information from the application documents. All patients, whether assigned to common medical rehabilitation or BMR, were then contacted by the researchers before the rehabilitation programme started and asked to complete the questionnaires. Individuals were excluded if they received neither a common rehabilitation programme nor a BMR programme. Further exclusion criteria were: people with an unknown employment status, incomplete information on the duration of rehabilitation or treatment dose delivered, and those who started their rehabilitation too late to ensure that the 10-month follow-up assessment was feasible.

### Outcomes and other measures

#### Primary outcome

The primary outcome was a stable return to work 10 months after rehabilitation. Stable return to work was defined as at least 4 weeks of employment without sick leave according to Kuijer et al. (16).

#### Secondary outcomes

As secondary outcomes, various health, work and coping outcomes were assessed. These were days of sick leave, current sick leave, self-rated work ability (17), general health (18, 19), physical functioning (20), depression (21), anxiety (22), pain intensity and pain disability (23, 24), fear-avoidance beliefs (25, 26), self-management skills (27), use of medications (pain medications, antidepressants, other medications), perceived risk of permanent work disability (28), and current unemployment. Higher scores in self-rated work ability, general health, physical functioning and self-management skills indicated better outcomes, whereas higher scores for the other variables represented worse outcomes. In addition, treatment satisfaction was assessed (29). A detailed description of all measures is given in the study protocol of the primary study (15). In addition, administrative data on disability pensions until the end of 2017, employment days, as well as the duration of receiving unemployment benefits and sickness absence benefits, were provided by the Federal German Pension Insurance.

#### Dose delivered

In order to check guideline fidelity, the dose delivered for the psychological therapy group, individual psychological consultations, relaxation training, exercise group and initial and final consultation in exercise therapy were assessed. Furthermore, the dose of the single BMR modules was summed to determine the total BMR treatment dose. The dose delivered was extracted from the administrative records.

#### Covariates

The following additional variables were only assessed at baseline before the start of rehabilitation to consider them when calculating the propensity scores: sociodemographic characteristics (sex, age, educational level, partnership, number of children and first language), somatization (30), pain generalization, psychosocial strain (31), type of musculoskeletal disorder, number of comorbidities, mental health comorbidity, the intention to deal with work-related problems during the rehabilitation programme (32), characteristics of the employment situation (employment status, sickness absence within the preceding 12 months, current sick leave, employment position and economic sector) and the risk of failing to return to work (33).

### Statistical analysis

For characterization of the samples in both treatment groups, descriptive statistics were used. To achieve an unbiased comparison of patients treated in BMR or common medical rehabilitation, for each patient treated in BMR, 1 similar person was identified from the larger pool of individuals treated in common medical rehabilitation. Matching was achieved using propensity scores (nearest-neighbour matching without replacement). The propensity score is the conditional probability to receive the treatment, i.e. BMR instead of a common medical rehabilitation programme (34). This probability was calculated with a logistic regression model using the following 32 variables: sex, age, educational level, partnership, employment status, economic sector, employment position, days of sick leave within the preceding 12 months, current sick leave, self-rated work ability, perceived risk of permanent work disability, intention to deal with workrelated problems during the rehabilitation programme, chronic back pain, number of secondary comorbidities, mental health comorbidity, use of pain medications, use of antidepressants, use of other medications, first language, number of children, general state of health, physical functioning, depression, anxiety, risk of failing to return to work, pain intensity, pain disability, pain generalization, somatization, fear-avoidance beliefs, psychosocial strain and self-management skills. Their inclusion in the logistic regression model was driven by the idea of considering the most relevant factors that may be associated with both treatments and outcomes. The propensity scores were calculated from 5 multiple imputed data-sets and then averaged. Multiple imputation was chosen to avoid the reduction in sample size when modelling the propensity scores. The propensity scores were estimated separately in the 5 data sets and then averaged (35).

The matched control group had similar characteristics to the group of BMR patients, thus allowing an unbiased estimation of the treatment effect for patients who were actually treated in BMR. Linear and logistic regression models were used to compare the outcomes of both treatment groups. Median time to return to work was estimated using a parametric regression survival-time model. Random-effect models were used because participants were clustered within centres. In addition, standardized treatment effects were calculated using Cohen’s d (36). Adjusted predicted estimates and standard errors were reported for each group, together with the absolute differences between BMR and common medical rehabilitation as well as their 95% CIs.

The results of the statistical tests were regarded as significant if the 2-sided *p*-value of a test was less than 0.05. All calculations were performed in Stata SE 15.0.

## RESULTS

### Recruitment and participants

**Fig. 1:**
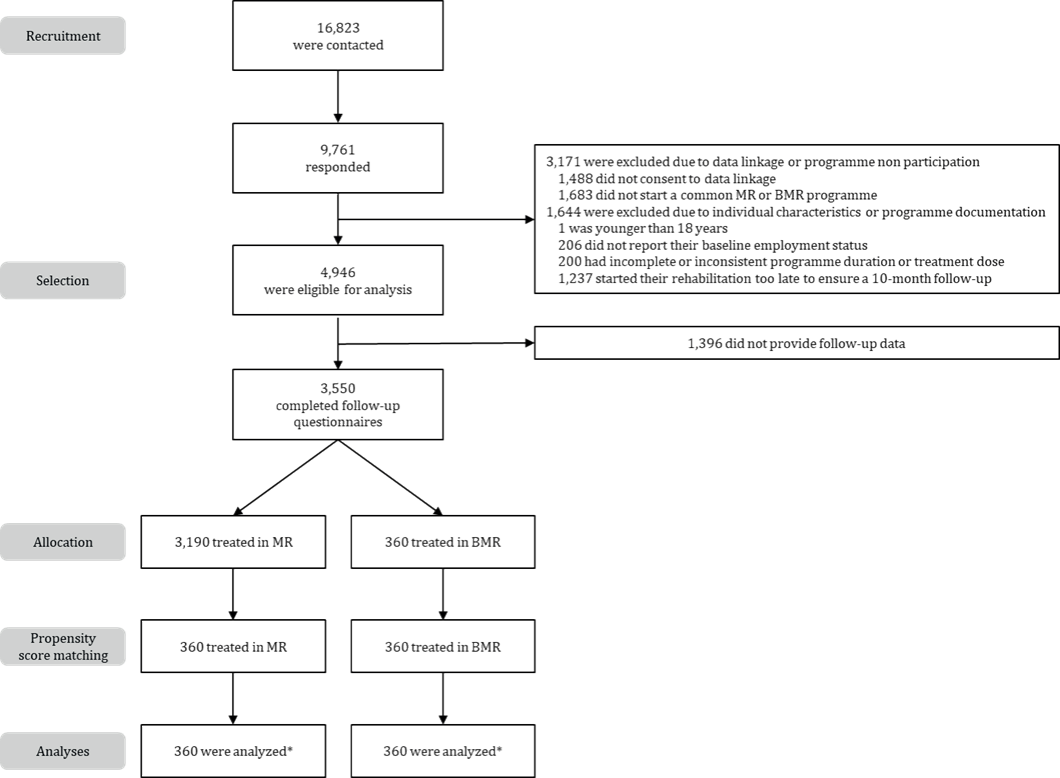
Flow of participants. *Number of cases included when estimating treatment effects varied due to missing values. MR: medical rehabilitation; BMR: behavioural medical rehabilitation.

A total of 16,823 patients who had been approved for rehabilitation due to musculoskeletal disorders were contacted by post. A total of 9,761 patients completed the baseline questionnaire between March and July 2016 before starting their programme. Of these, 3,171 patients were excluded as they did not consent to linkage of the questionnaire and administrative data, or they had not started a common medical rehabilitation or a BMR programme. Another 1,644 patients were excluded because they were younger than 18 years of age or did not report their baseline employment status, the duration of the rehabilitation programme or their treatment dose was incomplete or inconsistent, or they started the rehabilitation too late to ensure the follow-up at 10 months. Finally, 3,550 patients (72%) completed both the 3-month and the 10-month follow-up questionnaires. Of these, 3,190 patients were treated in a common medical rehabilitation programme and 360 in a BMR programme. The final matched sample included 720 patients, with 360 in each arm. Details of the full participant flow according to TREND are shown in Fig. 1.

### Baseline characteristics

The mean age of study participants was approximately 53.5 years (SD 7.0) and 74.0% were women. In addition to the primary musculoskeletal disorder, 22% of the total sample was diagnosed with a condition from the spectrum of mental health and behavioural disorders (International Statistical Classification of Diseases and Related Health Problems, 10^th^ Revision, German Modification (ICD-10-GM)-2019: F00–F99). Overall, 39% of all participants reported increased depression scores (Patient Health Questionnaire-2 (PHQ–2) ≥3) and 35% reported increased anxiety scores (Generalized Anxiety Disorder Scale-2 (GAD–2) ≥3) before rehabilitation. The proportion of missing values in the baseline varied between 0 and 4.8%. The samples of participants in BMR and the common medical rehabilitation programme were balanced for all of the variables that were included when calculating the propensity scores (Table I and Table SI).

**Table I:** Baseline characteristics

	Behavioural medical rehabilitation (*n* = 360)			Medical rehabilitation (*n* = 3,190)			Matched medical rehabilitation (*n* = 360)		
	*n*	%	Mean (SD)	*n*	%	Mean (SD)	*n*	%	Mean (SD)
Sex									
Female	283	78.6		2,353	73.8		285	79.2	
Male	77	21.4		837	26.2		75	20.8	
Age (years)	360		54.1 (6.4)	3,190		53.5 (7.0)	360		54.0 (6.1)
Educational level									
Low	86	24.0		736	23.2		92	25.7	
Average	216	60.2		1,893	59.6		210	58.7	
High	57	15.9		549	17.3		56	15.6	
Missing	1			12			2		
Employment									
Employed	347	96.4		3,051	95.6		347	96.4	
Unemployed	13	3.6		139	4.4		13	3.6	
Sick leave during the last 12 months (weeks)	356		8.1 (10.2)	3,116		8.4 (10.7)	354		7.5 (10.8)
Mental health comorbidity									
Yes	195	54.2		577	18.1		196	54.4	
No	165	45.8		2,613	81.9		164	45.6	
General health (0–10)	356		4.6 (1.7)	3,134		4.6 (1.7)	353		4.7 (1.8)
Physical functioning (0–10)	360		4.7 (1.8)	3,180		4.3 (1.8)	360		4.7 (1.8)
Depression (0–6)	359		2.9 (1.5)	3,162		2.4 (1.5)	358		2.8 (1.4)
Anxiety (0–6)	359		2.8 (1.5)	3,171		2.1 (1.6)	359		2.8 (1.6)
Pain intensity (0–100)	355		59.5 (17.7)	3,172		60.2 (17.2)	358		59.8 (17.8)
Pain disability (0–100)	353		53.3 (19.1)	3,148		55.8 (19.3)	354		53.2 (20.1)
Somatization (0–27)	336		7.0 (4.2)	3,077		5.8 (3.9)	351		6.9 (4.0)
Fear-avoidance beliefs (0–10)	351		4.0 (2.5)	3,147		4.5 (2.5)	356		3.7 (2.5)
Private/occupational strains (0–6)	353		3.7 (1.4)	3,162		2.9 (1.6)	355		3.7 (1.5)
Self-management skills (0–9)	357		4.1 (1.8)	3,151		4.5 (1.8)	354		4.1 (1.8)

The complete table of all baseline characteristics is available in Table SI.

*n* = 3,550; medical rehabilitation: n=3.190; behavioural medical rehabilitation: *n* = 360.

Sample size varies due to cases with missing data. In case of categorical variables, deviations from 100% are due to rounding.

### Dose delivered

The treatment dose of the behavioural medical treatment components was almost in accordance with the guidelines. On average, the recommended treatment dose was only achieved for the individual psychological consultations and the relaxation training (Table II). However, deviations from the recommendations were small, apart from initial and final consultation in exercise therapy. Patient satisfaction was comparable in both groups (BMR: mean 25.5 (SD 0.3); MR: mean 25.3 (SD 0.3); *p* = 0.629).

**Table II:** Implementation of behavioural medical treatment components

Components	*n*	Adjusted predicted estimates with SE		Difference	95% CI	*p*-value
		BMR	MR			
Psychological therapy group (min per rehabilitation)	720	422.47 (30.34)	136.38 (13.99)	286.09	220.61 to 351.57	< 0.001
Individual psycho-logical consultations (min per rehabilitation)	720	116.94 (10.19)	48.18 (5.14)	68.76	46.39 to 91.13	< 0.001
Relaxation training (min per rehabilitation)	720	254.20 (20.68)	175.26 (9.83)	78.95	34.06 to 123.83	0.001
Exercise group (min per rehabilitation)	720	1,149.90 (108.32)	742.78 (46.52)	407.12	176.07 to 638.18	0.001
Consultations in exercise therapy (min per rehabilitation)	720	9.90 (52.83)	42.35 (17.85)	–32.45	–141.74 to 76.85	0.561
Treatment intensity (min/day)	720	75.37 (5.64)	49.26 (2.28)	26.11	14.20 to 38.03	< 0.001
Total BMR treatment dose (h)	720	32.53 (2.13)	18.91 (0.91)	13.63	9.08 to 18.17	< 0.001

Analyses are based on the matched samples of 360 behavioural medical rehabilitation (BMR) patients and 360 similar patients treated in a common medical rehabilitation (MR).

### Primary and secondary outcomes

Ten months after the end of rehabilitation, no treatment effect in favour of BMR was found for the primary outcome, stable return to work. However, participants in BMR reported better results across a range of secondary outcomes (Table III). Self-reported work ability increased by 0.48 points (Cohen’s *d* = 0.19; 95% CI = 0.02, 0.35), physical functioning improved by 0.28 points (*d* =0.13; 95% CI = 0.00, 0.26) and self-management skills by 0.41 points (*d* =0.22; 95% CI = 0.08, 0.35). Pain disability decreased by 3.90 points (*d* =–0.17; 95% CI = –0.31, –0.03) and fear-avoidance beliefs by 0.45 points (*d* = 0.18; 95% CI = –0.33, –0.03).

**Table III:** Primary and secondary outcomes

Outcomes	*n*	Adjusted predicted estimates with SE		Difference	95% CI	*p*-value
		BMR	MR			
Primary outcome						
Stable return to work^a^	712	0.87 (0.02)	0.88 (0.02)	–0.01	–0.06 to 0.04	0.758
*Secondary outcomes*						
Time to return to work (days median)	709	5.26 (1.51)	4.12 (0.83)	1.13	–2.24 to 4.51	0.510
Work Ability Score (0–10)	702	6.45 (0.17)	5.96 (0.14)	0.48	0.05 to 0.91	0.028
General health (0–10)	695	5.92 (0.10)	5.71 (0.10)	0.21	–0.07 to 0.50	0.144
Physical functioning (0–10)	718	5.67 (0.11)	5.39 (0.09)	0.28	0.00 to 0.57	0.050
Pain intensity (0–100)	708	48.26 (1.20)	50.65 (1.02)	–2.40	–5.49 to 0.69	0.129
Pain disability (0–100)	697	40.75 (1.27)	44.65 (1.12)	–3.90	–7.21 to –0.59	0.021
Depression (0–6)	712	2.00 (0.07)	2.14 (0.07)	–0.14	–0.34 to 0.06	0.164
Anxiety (0–6)	713	2.06 (0.09)	2.13 (0.07)	–0.06	–0.29 to 0.16	0.567
Fear-avoidance beliefs (0–10)	698	3.36 (0.14)	3.81 (0.13)	–0.45	–0.82 to –0.09	0.016
Self-management skills (0–9)	703	5.84 (0.09)	5.42 (0.09)	0.41	0.15 to 0.67	0.002
Frequent use of pain medication^a^	715	0.52 (0.03)	0.53 (0.03)	–0.01	–0.08 to 0.06	0.823
Frequent use of antidepressants^a^	714	0.04 (0.01)	0.04 (0.01)	–0.00	–0.04 to 0.03	0.848
Frequent use of other medications^a^	711	0.19 (0.02)	0.21 (0.02)	–0.01	–0.07 to 0.05	0.687
Perceived risk of permanent work disability (0–3)	654	1.12 (0.06)	1.13 (0.05)	–0.01	–0.16 to 0.14	0.930
Unemployment^a^	714	0.09 (0.02)	0.10 (0.02)	–0.01	–0.06 to 0.03	0.626
Employment days^b^	671	288.87 (9.48)	292.33 (7.64)	–3.46	–27.32 to 20.40	0.776
Days with unemployment benefits^b^	671	11.38 (3.42)	22.10 (3.42)	–10.72	–20.21 to –1.24	0.027
Days with sickness absence benefits^b^	671	26.47 (3.50)	20.39 (3.51)	6.08	–3.64 to 15.80	0.220
Disability pension requests^a,b^	720	0.05 (0.02)	0.05 (0.01)	0.00	–0.04 to 0.04	0.888

Analyses are based on the matched samples of 360 behavioural medical rehabilitation (BMR) patients and 360 similar patients treated in a common medical rehabilitation (MR). Sample size varies due to cases with missing data.

a Binary outcome.

b In the year after the rehabilitation.

No significant differences were found for the number of disability pension requests, employment days and days of sickness absence benefits in the year after the rehabilitation between the 2 groups. However, days with unemployment benefits in the year after the rehabilitation were less after a BMR (*d* = –0.17; 95% CI = –0.32, –0.02).

## DISCUSSION

Ten months after rehabilitation, no difference was detected regarding return to work between participants in a BMR programme and those in a MR programme. However, several other effects were observed in favour of BMR. Ten months after rehabilitation, participants in BMR reported better self-reported work ability than comparable participants in a MR programme. In addition, they reported increased physical functioning and self-management skills, decreased pain disability and fear-avoidance beliefs and fewer days with unemployment benefits. However, these effects were small. No clinically meaningful effect of BMR regarding disability pension requests was found. Participants in BMR, however, had fewer days receiving unemployment benefits in the year after the rehabilitation.

The results of this study on the effectiveness of BMR in real-world care are similar to those of previous studies. Participation in MR, with combined pain competency and depression prevention training, also resulted in better self-reported work ability, measured with the Work Ability Index in a former randomized controlled trial (11, 17). The Work Ability Index is strongly correlated with the Work Ability Score used in our study (37). Contrary to previous findings (10), the current study found no effects in favour of BMR on pain intensity 10 months after rehabilitation. However, participants in BMR reported less pain disability in the current study. This indicates improved pain management strategies, which were also improved in favour of BMR in previous randomized controlled trials (10). Despite these positive effects, there were no statistically significant benefits of BMR in comparison with MR with regard to disability pension requests, receipt of sickness absence benefits, or employment in the year after the rehabilitation.

Analyses of the treatment dose delivered showed that BMR has not yet been completely implemented. However, treatment adherence is a key condition for the effectiveness of medical programmes. Deviations from the recommended treatment dose and dose variations between rehabilitation centres are normal when implementing new complex interventions (38). Since the implementation of new programmes often involves new skills and hiring of additional staff, rehabilitation centres are usually prepared differently for the implementation challenge. Some institutions were involved in earlier randomized controlled trials, accompanied by the development of guidelines and were therefore well prepared for the guideline recommendations. Further support for the implementation should take these differences into account.

When interpreting these findings, the following limitations must be considered. First, although propensity score matching is a powerful tool to reduce bias, a propensity score matched analysis is still based on observational data only (39). It is not a randomized controlled trial. Consequently, the results are potentially biased by unobserved differences between patients treated in BMR and in common medical rehabilitation. Secondly, selection bias due to selective non-participation and selective follow-up non-responses might have biased our estimates. Thirdly, the systematic selection of the participants in the MR was carried out in such a way that only participants were considered who were similar to the participants in the BMR. The differences between BMR and MR described here apply exclusively to the patients treated in BMR (“average treatment effect for the treated”) (40). Fourthly, therapy concepts may have differed across rehabilitation centres, as psychological therapy concepts, in particular, vary depending on the orientation of the centre and the psychotherapists treating the patient. However, we cannot describe these qualitative differences using the data collected in the current study. Fifthly, analysis of the treatment dose delivered reflected only the treatments documented in the discharge letters by quantity, but not by content and quality. Furthermore, the analysis cannot exclude the fact that the overall longer duration of BMR caused slightly better outcomes in favour of BMR. Sixthly, in only 10 out of 92 rehabilitation centres BMR was provided. Thus, the 360 patients treated in MR are spread across 82 rehabilitation centres. Therefore, there may be other contributing factors that are not related to the treatment programme, but to the rehabilitation centre (e.g. more therapeutic staff or higher budget).

The limitations of the study are balanced by the following strengths. First, the sample size was large enough to realize propensity score matching without sample losses in the group of BMR participants. For the estimation of propensity scores, administrative data and data from the self-assessment questionnaires were used. In addition, propensity score matching reduced the different initial conditions to a minimum, and balanced sample characteristics could be achieved (34). Secondly, a rigorous approach was used to check guideline fidelity. This provided a valuable insight into how implementation can be improved. Thirdly, the large number of rehabilitation centres in MR represents a reliable representation of conventional medical rehabilitation in Germany. Fourthly, our pragmatic effectiveness trial tested the effects of a new rehabilitation approach in a real-world care environment. We believe that implementation research should document fidelity, check whether the anticipated goals are also achieved in real-world care settings, and assess the degree to which findings from efficacy studies can be replicated.

In conclusion, compared with common MR, BMR leads to better self-reported work ability, better physical functioning, less pain disability, fewer fear-avoidance beliefs and better self-management skills 10 months after rehabilitation. The results indicate that BMR is also effective under real-life conditions regarding pain and disease management skills. However, the beneficial effects are small and there is no effect on return to work. At the same time, it should be noted that BMR is not inferior to common MR. It is necessary to examine whether a more consistent implementation of the BMR guideline offers the opportunity to improve outcomes in real-life care.

## Supplementary Material



## References

[1] Woolf AD, Erwin J, March L. The need to address the burden of musculoskeletal conditions. Best Pract Res Clin Rheumatol 2012; 26: 183–224. 22794094 10.1016/j.berh.2012.03.005

[2] Schmidt CO, Raspe H, Pfingsten M, Hasenbring M, Basler HD, Eich W, et al. Back pain in the German adult population: prevalence, severity, and sociodemographic correlates in a multiregional survey. Spine 2007; 32: 2005–2011. 17700449 10.1097/BRS.0b013e318133fad8

[3] Deutsche Rentenversicherung Bund. Rentenversicherung in Zeitreihen 2019. [Pension insurance in time series 2019.] Berlin: Deutsche Rentenversicherung Bund; 2019 (in German).

[4] Schmidt C, Bernert S, Spyra K. Zur Relevanz psychischer Komorbiditäten bei chronischem Rückenschmerz: Häufigkeitsbild, Erwerbsminderungsrenten und Reha-Nachsorge im Zeitverlauf der Reha-Kohorten 2002–2009. [Concerning the impact of psychological comorbidity for chronic back pain: frequency, reduced earning capacity pension and rehabilitation aftercare in the course of the rehabilitation cohorts 2002–2009.] Rehabilitation 2014; 53: 384–389 (in German). 25494344 10.1055/s-0034-1394449

[5] Aartun E, Axén I, Mior S, Røe Y, Hondras M, Kretz L, et al. Contextualizing the lived experiences of patients with low back pain from different countries according to the ICF framework. J Rehabil Med 2021; 53: jrm00189. 33778896 10.2340/16501977-2819 PMC8814864

[6] Deutsche Rentenversicherung Bund. Positionspapier der Deutschen Rentenversicherung zur Bedeutung psychischer Erkrankungen in der Rehabilitation und bei Erwerbsminderung. [Position paper of the German Pension Insurance on the importance of mental disorders in rehabilitation and in work disability.] Berlin: Deutsche Rentenversicherung Bund; 2014 (in German).

[7] Deutsche Rentenversicherung Bund. Anforderungsprofil der Deutschen Rentenversicherung Bund für die verhaltensmedizinisch orientierte Rehabilitation (VOR). [Guideline for behavioral medical rehabilitation (BMR) by the Federal German Pension Insurance.] 2nd edn. Berlin: Deutsche Rentenversicherung Bund; 2016 (in German).

[8] Mangels M, Schwarz S, Worringen U, Holme M, Rief W. Evaluation of a behavioral-medical inpatient rehabilitation treatment including booster sessions: a randomized controlled study. Clin J Pain 2009; 25: 356–364. 19454868 10.1097/AJP.0b013e3181925791

[9] Hampel P, Tlach L, Gräf T, Krohn-Grimberghe B, Mantel F, Mohr B. Zur Wirksamkeit eines Trainings zur Depressionsbewältigung für Patienten mit chronisch unspezifischem Rückenschmerz in der stationären Rehabilitation – Eine 1-Jahres-Follow up-Studie. [On the effectiveness of depression coping training for patients with chronic nonspecific back pain in inpatient rehabilitation – a 1-year follow-up study.] DRV-Schriften 2009; 83: 322–325 (in German).

[10] Bethge M, Müller-Fahrnow W. Wirksamkeit einer intensivierten stationären Rehabilitation bei muskuloskelettalen Erkrankungen: systematischer Review und Meta-Analyse. [Efficacy of intensified inpatient rehabilitation in musculoskeletal disorders: systematic review and meta-analysis.] Rehabilitation 2008; 47: 200–209 (in German). 18704869 10.1055/s-2008-1077091

[11] Hampel P, Köpnick A, Roch S. Psychological and work-related outcomes after inpatient multidisciplinary rehabilitation of chronic low back pain: a prospective randomized controlled trial. BMC Psychol 2019; 7: 6. 30770763 10.1186/s40359-019-0282-3 PMC6377771

[12] Glasgow RE, Lichtenstein E, Marcus AC. Why don’t we see more translation of health promotion research to practice? Rethinking the efficacy-to-effectiveness transition. Am J Public Health 2003; 93: 1261–1267. 12893608 10.2105/ajph.93.8.1261 PMC1447950

[13] Des Jarlais DC, Lyles C, Crepaz N. Improving the reporting quality of nonrandomized evaluations of behavioral and public health interventions: the TREND statement. Am J Public Health 2004; 94: 361–366. 14998794 10.2105/ajph.94.3.361 PMC1448256

[14] Bethge M, Markus M, Streibelt M, Gerlich C, Schuler M. Effects of nationwide implementation of work-related medical rehabilitation in Germany: propensity score matched analysis. Occup Environ Med 2019; 76: 913–919. 31594839 10.1136/oemed-2019-106065

[15] Neuderth S, Schwarz B, Gerlich C, Schuler M, Markus M, Bethge M. Work-related medical rehabilitation in patients with musculoskeletal disorders: the protocol of a propensity score matched effectiveness study (EVA-WMR, DRKS00009780). BMC Public Health 2016; 16: 804. 27534527 10.1186/s12889-016-3437-7 PMC4989326

[16] Kuijer PP, Gouttebarge V, Wind H, van Duivenbooden C, Sluiter JK, Frings-Dresen MH. Prognostic value of self-reported work ability and performance-based lifting tests for sustainable return to work among construction workers. Scand J Work Environ Health 2012; 38: 600–603. 22538928 10.5271/sjweh.3302

[17] Ilmarinen J. The Work Ability Index (WAI). Occup Med 2007; 57: 160.

[18] Kristensen TS, Hannerz H, Hogh A, Borg V. The Copenhagen Psychosocial Questionnaire - a tool for the assessment and improvement of the psychosocial work environment. Scand J Work Environ Health 2005; 31: 438–449. 16425585 10.5271/sjweh.948

[19] Nuebling M, Hasselhorn HM. The Copenhagen Psychosocial Questionnaire in Germany: from the validation of the instrument to the formation of a job-specific database of psychosocial factors at work. Scand J Public Health 2010; 38: 120–124. 21172777 10.1177/1403494809353652

[20] Wirtz M, Farin E, Bengel J, Jäckel WH, Hammerer D, Gerdes N. IRES-24 patient questionnaire: development of the short form of an assessment instrument in rehabilitation by means Mixed-Rasch analysis. Diagnostica 2005; 51: 75–87.

[21] Lowe B, Kroenke K, Grafe K. Detecting and monitoring depression with a two-item questionnaire (PHQ-2). J Psychosom Res 2005; 58: 163–171. 15820844 10.1016/j.jpsychores.2004.09.006

[22] Kroenke K, Spitzer RL, Williams JB, Lowe B. The Patient Health Questionnaire Somatic, Anxiety, and Depressive Symptom Scales: a systematic review. Gen Hosp Psychiatry 2010; 32: 345–359. 20633738 10.1016/j.genhosppsych.2010.03.006

[23] Von Korff M, Ormel J, Keefe FJ, Dworkin SF. Grading the severity of chronic pain. Pain 1992; 50: 133–149. 1408309 10.1016/0304-3959(92)90154-4

[24] Klasen BW, Hallner D, Schaub C, Willburger R, Hasenbring M. Validation and reliability of the German version of the Chronic Pain Grade questionnaire in primary care back pain patients. Psychosoc Med 2004; 1: Doc07. 19742049 PMC2736479

[25] Pfingsten M, Kroner-Herwig B, Leibing E, Kronshage U, Hildebrandt J. Validation of the German version of the Fear-Avoidance Beliefs Questionnaire (FABQ). Eur J Pain 2000; 4: 259–266. 10985869 10.1053/eujp.2000.0178

[26] Kent P, Mirkhil S, Keating J, Buchbinder R, Manniche C, Albert HB. The concurrent validity of brief screening questions for anxiety, depression, social isolation, catastrophization, and fear of movement in people with low back pain. Clin J Pain 2014; 30: 479–489. 24281277 10.1097/AJP.0000000000000010

[27] Osborne RH, Elsworth GR, Whitfield K. The Health Education Impact Questionnaire (heiQ): an outcomes and evaluation measure for patient education and self-management interventions for people with chronic conditions. Patient Educ Couns 2007; 66: 192–201. 17320338 10.1016/j.pec.2006.12.002

[28] Mittag O, Raspe H. Eine kurze Skala zur Messung der subjektiven Prognose der Erwerbstätigkeit: Ergebnisse einer Untersuchung an 4279 Mitgliedern der gesetzlichen Arbeiterrentenversicherung zu Reliabilität (Guttman-Skalierung) und Validität der Skala. [A brief scale for measuring subjective prognosis of gainful employment: findings of a study of 4279 statutory pension insurees concerning reliability (Guttman Scaling) and validity of the scale.] Rehabilitation 2003; 42: 169–174 (in German). 12813654 10.1055/s-2003-40095

[29] Attkisson CC, Zwick R. The client satisfaction questionnaire. Psychometric properties and correlations with service utilization and psychotherapy outcome. Evaluat Prog Plan 1982; 5: 233–237. 10.1016/0149-7189(82)90074-x 10259963

[30] Schmitz N, Hartkamp N, Kiuse J, Franke GH, Reister G, Tress W. The Symptom Check-List-90-R (SCL-90-R): a German validation study. Qual Life Res 2000; 9: 185–193. 10983482 10.1023/a:1008931926181

[31] Küch D, Arndt S, Grabe A. UKS – Ultrakurzscreening psychosozialer Problemlagen zur bedarfsorientierten Angebotszuweisung in der somatischen Rehabilitation. [UKS - Ultra brief screening of psychosocial problems for needsoriented service allocation in somatic rehabilitation.] Bonn: Deutscher Psychologen Verlag, 2011 (in German).

[32] Lukasczik M, Wolf H-D, Gerlich C, Löffler S, Vogel H, Faller H, et al. Current state of vocationally oriented medical rehabilitation – a German perspective. Disabil Rehabil 2011; 33: 2646–2655. 21539472 10.3109/09638288.2011.575528

[33] Streibelt M, Bethge M. Prospective cohort analysis of the predictive validity of a screening instrument for severe restrictions of work ability in patients with musculoskeletal disorders. Am J Phys Med Rehabil 2015; 94: 617–626. 25299532 10.1097/PHM.0000000000000220

[34] Austin PC. An introduction to propensity score methods for reducing the effects of confounding in observational studies. Multivariate Behav Res 2011; 46: 399–424. 21818162 10.1080/00273171.2011.568786 PMC3144483

[35] Mitra R, Reiter JP. A comparison of two methods of estimating propensity scores after multiple imputation. Stat Methods Med Res 2016; 25: 188–204. 22687877 10.1177/0962280212445945

[36] Cohen J. Statistical power analysis for the behavioral sciences. Hillsdale: L. Erlbaum Associates; 1988.

[37] El Fassi M, Bocquet V, Majery N, Lair ML, Couffignal S, Mairiaux P. Work ability assessment in a worker population: comparison and determinants of Work Ability Index and Work Ability Score. BMC Public Health 2013; 13: 305. 23565883 10.1186/1471-2458-13-305 PMC3637198

[38] Damschroder LJ, Aron DC, Keith RE, Kirsh SR, Alexander JA, Lowery JC. Fostering implementation of health services research findings into practice: a consolidated framework for advancing implementation science. Implement Sci 2009; 4: 50. 19664226 10.1186/1748-5908-4-50 PMC2736161

[39] Schelvis RM, Oude Hengel KM, Burdorf A, Blatter BM, Strijk JE, van der Beek AJ. Evaluation of occupational health interventions using a randomized controlled trial: challenges and alternative research designs. Scand J Work Environ Health 2015; 41: 491–503. 26030719 10.5271/sjweh.3505

[40] Imbens GW. Nonparametric estimation of average treatment effects under exogeneity: a review. Rev Econ Stat 2004; 86: 4–29.

