# Supplementary material for: EFFECTIVENESS OF BEHAVIOURAL MEDICAL REHABILITATION UNDER REALLIFE CONDITIONS IN GERMANY: A PROPENSITY-SCORE MATCHED ANALYSIS
Source: J Rehabil Med. 2021 Oct 21;54:469. doi: 10.2340/16501977-jrm.v53.469 (PMC8862651; doi:10.2340/16501977-jrm.v53.469)
Supplement: Supplementary file 1 [file JRM-54-469-s1.pdf]

Supplementary material to article by M. Markus et al. "Effectiveness of behavioural medical rehabilitation under real-life conditions in germany: a propensity-score matched analysis"

Table SI. Complete baseline characteristics

	Behavioural medical rehabilitation ($n=360$)			Medical rehabilitation ($n=3,190$)			Matched medical rehabilitation ($n=360$)		
	n	%	Mean (SD)	*n*	%	Mean (SD)	*n*	%	Mean (SD)
Sex									
Female	283	78.6		2,353	73.8		285	79.2	
Male	77	21.4		837	26.2		75	20.8	
Age, years	360		54.1 (6.4)	3,190		53.5 (7.0)	360		54.0 (6.1)
Educational level									
Low	86	24.0		736	23.2		92	25.7	
Average	216	60.2		1,893	59.6		210	58.7	
High	57	15.9		549	17.3		56	15.6	
Missing	1			12			2		
Partnership									
Yes	265	74.6		2,474	78.6		265	74.6	
No	90	25.4		674	21.4		90	25.4	
Missing	5			42			5		
Employment									
Employed	347	96.4		3,051	95.6		347	96.4	
Unemployed	13	3.6		139	4.4		13	3.6	
Economic sector									
Industry	50	14.5		423	13.9		45	13.1	
Craft	15	4.3		160	5.3		10	2.9	
Service	173	50.1		1,442	47.5		182	52.9	
Public service	40	11.6		456	15.0		41	11.9	
Other	67	19.4		555	18.3		66	19.2	
Missing	15			154			16		
Employment position									
Blue-collar worker	320	89.4		2,851	90.0		323	90.0	
White-collar worker	38	10.6		316	10.0		36	10.0	
Missing	2			23			1		
Sick leave during the last 12 months (weeks)	356		8.1 (10.2)	3,116		8.4 (10.7)	354		7.5 (10.8)
Current sick leave									
Yes	68	18.9		820	26.0		61	17.1	
No	291	81.1		2,335	74.0		296	82.9	
Missing	1			35			3		
Work Ability Score (0–10)	356		5.0 (2.2)	3,149		5.0 (2.4)	356		5.0 (2.3)
Perceived risk of permanent work disability (0–3)	340		1.2 (1.0)	3,046		1.1 (1.0)	349		1.3 (1.0)
Intention to deal with work-related problems (1–5)	353		3.5 (1.3)	3,138		2.9 (1.5)	354		3.5 (1.4)
Chronic back pain (ICD–10)*									
Yes	345	95.8		2,833	88.8		345	95.8	
No	15	4.2		357	11.2		15	4.2	
Number of comorbidities									
0	1	0.3		153	4.8		0	0.0	
1	28	7.8		396	12.4		31	8.6	
2	53	14.7		630	19.7		53	14.7	
3	78	21.7		652	20.4		84	23.3	
4	200	55.6		1.359	42.6		192	53.3	
Mental health comorbidity									
Yes	195	54.2		577	18.1		196	54.4	
No	165	45.8		2,613	81.9		164	45.6	
Frequent use of pain medication									
Yes	289	81.0		2,566	80.6		287	79.9	
No	68	19.0		617	19.4		72	20.1	
Missing	7			7			1		
Frequent use of antidepressants									
Yes	79	22.3		344	10.8		84	23.3	
No	276	77.7		2,833	89.2		276	76.7	
Missing	5			13			0		
Frequent use of other medications									
Yes	254	70.9		2,178	68.6		253	71.1	
No	104	29.1		997	31.4		103	28.9	
Missing	2			15			4		
First language									
German	344	95.6		3,064	96.1		347	96.4	
Other	16	4.4		125	3.9		13	3.6	
Missing	0			1			0		
Number of children									
None	67	18.7		617	19.4		67	18.7	
1	79	22.1		740	23.3		70	19.5	
2	160	44.7		1,370	43.1		158	44.0	
3 or more	52	14.5		452	14.2		64	17.8	
Missing	2			11			1		
General health (0–10)	356		4.6 (1.7)	3,134		4.6 (1.7)	353		4.7 (1.8)
Physical functioning (0–10)	360		4.7 (1.8)	3,180		4.3 (1.8)	360		4.7 (1.8)

Table SI. *cont.*

	Behavioural medical rehabilitation (*n* = 360)			Medical rehabilitation (*n* = 3,190)			Matched medical rehabilitation (*n* = 360)		
	n	%	Mean (SD)	*n*	%	Mean (SD)	*n*	%	Mean (SD)
Depression (0–6)	359		2.9 (1.5)	3,162		2.4 (1.5)	358		2.8 (1.4)
PHQ–2≥3									
Yes	194	54.0		1,192	37.7		170	47.2	
No	165	46.0		1,970	62.3		188	52.8	
Missing	1			28			2		
Anxiety (0–6)	359		2.8 (1.5)	3,171		2.1 (1.6)	359		2.8 (1.6)
GAD–2≥3									
Yes	193	53.8		1,041	32.8		175	48.7	
No	166	46.2		2,130	67.2		184	51.3	
Missing	1			19			1		
PHQ-4 (0–12)	358		5.7 (2.7)	3,152		4.5 (2.8)	357		5.6 (1.7)
SIMBO (0–100)	358		15.6 (19.3)	3,163		17.1 (20.6)	358		15.0 (19.9)
Pain intensity (0–100)	355		59.5 (17.7)	3,172		60.2 (17.2)	358		59.8 (17.8)
Pain disability (0–100)	353		53.3 (19.1)	3,148		55.8 (19.3)	354		53.2 (20.1)
Pain generalization (0–9)	350		4.8 (2.1)	3,122		4.5 (2.3)	353		4.7 (2.1)
Somatization (0–27)	336		7.0 (4.2)	3,077		5.8 (3.9)	351		6.9 (4.0)
Fear-avoidance beliefs (0–10)	351		4.0 (2.5)	3,147		4.5 (2.5)	356		3.7 (2.5)
Private/occupational strains (0–6)	353		3.7 (1.4)	3,162		2.9 (1.6)	355		3.7 (1.5)
Self-management skills (0–9)	357		4.1 (1.8)	3,151		4.5 (1.8)	354		4.1 (1.8)

*Chronic back pain includes all dorsopathies coded M40 to M54 according to ICD-10. Sample size varies due to cases with missing data. In case of categorical variables deviations from 100% are due to rounding. ICD-10: International Statistical Classification of Diseases and Related Health Problems 10th revision; PHQ: Patient Health Questionnaire; GAD: Generalized Anxiety Disorder; SIMBO: Screening-Instrument zur Feststellung des Bedarfs an medizinisch-beruflich orientierten Maßnahmen in der medizinischen Rehabilitation (screening questionnaire to determine need for work-related medical rehabilitation).

JRM Journal of Rehabilitation Medicine JRM Journal of Rehabilitation Medicine JRM
